# Sidedness is not a prognostic factor in an unselected cohort of patients with colon cancer but prognosis for caecal carcinoma is worse – A multivariate analysis of a large single institution database

**DOI:** 10.1007/s00384-023-04590-8

**Published:** 2024-02-13

**Authors:** Sigmar Stelzner, Matthias Mehdorn, Erik Puffer, Dorothea Bleyl, Thomas Kittner, Philipp Rhode, Ines Gockel, Soeren T. Mees

**Affiliations:** 1 Department of General and Visceral Surgery, Dresden-Friedrichstadt General Hospital, Teaching Hospital of the Technical University Dresden, Friedrichstr. 41, D-01067 Dresden, Germany; 2 Department of Visceral, Transplant, Thoracic, and Vascular Surgery, University Hospital of Leipzig, Liebigstr. 20, D-04103 Leipzig, Germany; 3 Department of Pathology, Dresden-Friedrichstadt General Hospital, Teaching Hospital of the Technical University Dresden, Friedrichstr. 41, D-01067 Dresden, Germany; 4 Department of Medical Oncology, Dresden-Friedrichstadt General Hospital, Teaching Hospital of the Technical University Dresden, Friedrichstr. 41, D-01067 Dresden, Germany; 5 Department of Radiology, Dresden-Friedrichstadt General Hospital, Teaching Hospital of the Technical University Dresden, Friedrichstr. 41, D-01067 Dresden, Germany

**Keywords:** Colon cancer, Sidedness, Curative surgery, Synchronous metastases, Prognosis

## Abstract

**Purpose:**

Sidedness has emerged as a prognostic factor for metastatic colorectal cancer treated with modern systemic therapies. This study investigates whether it is also relevant for an unselected patient cohort including all stages.

**Methods:**

All consecutive patients admitted with colon cancer between 1995 and 2018 were retrieved from an institution-held database. Patients were divided into two cohorts. The first cohort included patients without distant metastases who were able to undergo curative resection. The second cohort presented with distant metastases (stage IV). Potentially prognostic factors were subjected to multivariate Cox Regression analysis.

**Results:**

Overall, 1,606 patients met the inclusion and exclusion criteria. An R0-resection was achieved in 1,222 patients without distant metastases. Five-year cause-specific survival rate was 89.3% for this group. There was no difference between right- and left-sided cancers (88.2% vs. 90.1%, p = 0.220). However, prognosis of caecal carcinoma was significantly worse than that of all other sites combined (83.5% vs. 90.2%, p = 0.007). In multivariate analysis, pT-category, pN-category, grading, vascular invasion, emergency operation, adjuvant chemotherapy, and caecal carcinoma remained as independent prognostic factors. In the 384 patients with stage IV-disease, 3-year overall survival for right- vs. left-sided cancers differed only in univariate analysis (17.7% vs. 28.6%, p = 0.013).

**Conclusion:**

In non-metastatic colon cancer, location in the caecum is an independent prognostic factor. In unselected patients with stage IV colon cancer, sidedness was not found to be a prognostic factor. Differentiation into right- and left-sided tumors may be simplistic, and further studies on the biological behavior of different colonic sites are warranted.

**Supplementary Information:**

The online version contains supplementary material available at 10.1007/s00384-023-04590-8.

## Introduction

With the introduction of modern targeted therapies for metastatic colorectal cancer (CRC), interest in the different outcomes of right- and left-sided tumors has been renewed [1–3]. These differences could be attributed to different molecular marker profiles [4], pathways of tumor development [5], and immunoresponse of the tumors [6]. The division of CRC into right- and left-sided tumors was established with the border of the embryologic midgut to the hindgut just proximal to the splenic flexure serving as the anatomic landmark. In these studies, no subdivisions of the various colonic segments were made, and colon and rectal cancers were usually investigated as a single entity. However, the rectum has several anatomical and functional features that distinguish it from the colon [7].

The concept of sidedness in CRC may be evident from the viewpoint of systemic therapy aimed at tumor metastases. It remains unclear whether this concept is also relevant for tumors at the outset, especially for stage I-III tumors, which are usually amenable to straight forward surgical resection. Additionally, it is unclear, whether the various colonic segments within the right or left side behave homogeneously, as presumed. The prognosis of tumors of the transverse colon, including the splenic flexure and descending colon, and thus entailing components of both sides has been reported to be inferior to that of the remaining colon [8]. Furthermore, the introduction of complete mesocolic excision (CME) by Hohenberger et al. provided a sound embryological concept for colon cancer surgery with an impact on outcomes [9, 10]. All these considerations require an in-depth investigation of the prognoses of different colonic segments.

This study aimed to investigate a large surgical database with respect to prognostic factors focusing solely on colon cancers (i.e., up to > 16 cm from the anal verge as defined by the Union Internationale Contre le Cancer (UICC) [11]). We hypothesized that the prognosis would be different for right- and left-sided primary tumors, whereas the behavior of the segments within the sides would be homogenous.

## Patients and methods

### Patient selection

The prospective database of colorectal and anal carcinomas held at Dresden-Friedrichstadt General Hospital was searched for colon cancers treated between 1995 and 2018. All patients gave informed consent for data collection. According to national regulations, no formal approval of the responsible institutional review board was necessary for this kind of study.

Patients with histologically confirmed invasive colon adenocarcinomas were included. The exclusion criteria were tumors other than adenocarcinoma, carcinoma in situ, tumors without staging information, and carcinoma of the appendix. Colonic segments were documented according to the International Classification of Diseases for Oncology (ICD-O) [12]. In patients with synchronous colon cancers in different segments, the most advanced cancer was selected for segment assignment. Similarly, in tumors involving more than one segment, the main mass was decisive for allocation. The right side of the colon was defined as cancer of the caecum, ascending colon, hepatic flexure, or transverse colon, whereas the left side included cancers from the splenic flexure to the sigmoid colon. Time of treatment was divided into two periods: 1995–2006 and 2007–2018. These intervals were chosen because CME was fully implemented in our colorectal unit from 2007 onwards. We further investigated UICC stages I-III tumors separately from stage IV tumors. In stage I-III tumors, the approach involves curative resection with only very few exceptions. To focus on prognosis following curative surgery, we excluded patients with locally resected tumors, patients after neoadjuvant therapy, patients with incomplete (R1/2) resection, and in-hospital mortality. Regarding the group of stage IV tumors, all patients were included in the analysis.

### Investigated parameters

All potential prognostic factors available in the database were investigated considering essential and additional factors as proposed by Compton et al. [13], and high-risk factors as defined by the National Comprehensive Cancer Network (NCCN) [14]. Age was dichotomized at the median. In addition to comparing right- and left-sided tumors we investigated the prognosis of each tumor location against the remaining cohort. T- and N-categories were analyzed separately next to UICC-stages. Number of harvested lymph nodes was divided into < 12 and ≥ 12 as recommended by the UICC. Well and moderately differentiated carcinomas were compared with poorly differentiated and undifferentiated tumors. The cut-off for the longitudinal length of the specimen was chosen to be at 20 cm, offering the potential for a 10 cm safety margin in both directions as an oncologically adequate operation. Sex, time period, preoperative CEA (normal vs. elevated), lymphovascular and vascular invasion, emergency surgery, and adjuvant chemotherapy were also investigated. For stage IV tumors, the best available information was used to define the T- and N-categories. The number of harvested lymph nodes was omitted because of the large number of patients who did not undergo primary tumor resection. Similarly, there were too many missing values to examine lymphovascular and vascular invasion. Resection of the primary tumor (irrespective of metastasectomy), R-classification (comprising the primary tumor and the metastases; R0/1 vs. R2), and metastatic sites (liver/other/multiple) were also included.

Missing data were supplemented with multiple imputations.

### Statistical analysis

Follow-up was performed in a specialized outpatient clinic as previously described [15]. Patients without information on death or actual life status at the 5-year point or at the closing date of the study (30th September 2021) were considered lost to follow-up. Survival was analyzed using the Kaplan–Meier product limit method and compared with the log rank test. P-values were derived from tow-tailed tests and statistical significance was set at p < 0.05. For patients with stage I-III disease, the 5-year cause specific survival (CSS) was calculated from the date of surgery, and death with tumor was defined as an event. Patients who survived longer than 60 months were censored at this time point. Similarly, patients who died from other causes and those who were lost to follow-up were censored on the date of the last available life status. For the calculation of recurrence free survival (RFS), death of any cause or diagnosis of locoregional or distant tumor recurrence was taken as event, whichever occurred first. For patients with stage IV disease, the 3-year overall survival (OS) was calculated from the date of diagnosis. Death from any cause was considered as an event. Patients who survived longer than 3 years were censored at the 3-year threshold. Patients who were lost to follow-up within 3 years were also censored. Factors which displayed significant differences in the univariate analysis were included in the Cox regression analysis. Only UICC stages in stage I-III carcinoma were left out because of their direct interrelationship with pT- and pN-categories. The proportional hazards assumption was tested using log minus log plots. All statistical analysis were performed with SPSS^®^ version 29 (IBM Corp., Armonk, NY, USA).

## Results

### Patient population-overview

**Fig. 1:**
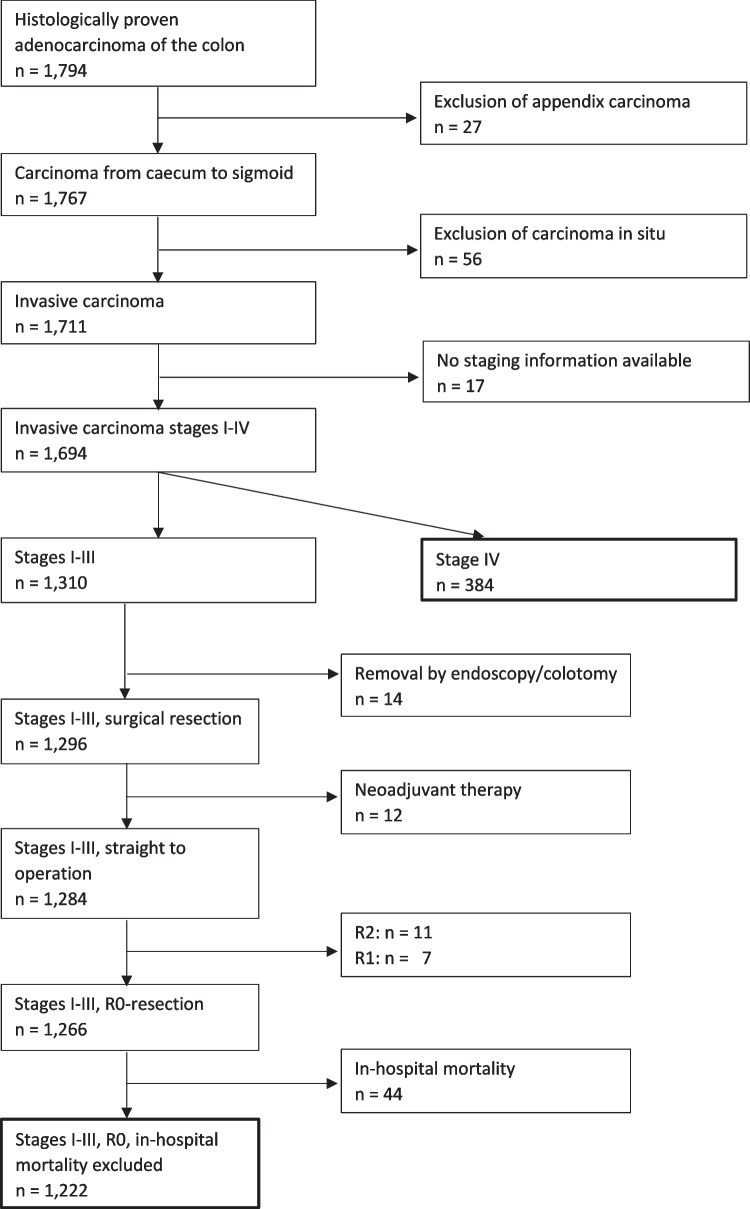
Flow-chart of the study population

Our database query retrieved 1,794 consecutive patients who were treated for histologically proven adenocarcinoma of the colon. After excluding appendiceal carcinoma (n = 27), carcinoma in situ (n = 56), and tumors without available staging information (n = 17), there remained 1,310 patients with stage I-III disease and 384 with synchronous metastases. While the latter were all included for further analysis, we excluded patients from the former group because of local resection (endoscopic or colotomy, n = 14), neoadjuvant treatment (n = 12), incomplete gross resection (R2, n = 11), incomplete microscopic resection (R1, n = 7), and in-hospital mortality (n = 44). Thus, n = 1,222 patients with stage I-III disease were further analyzed (Fig. 1).

### Stage I-III tumors

**Fig. 2:**
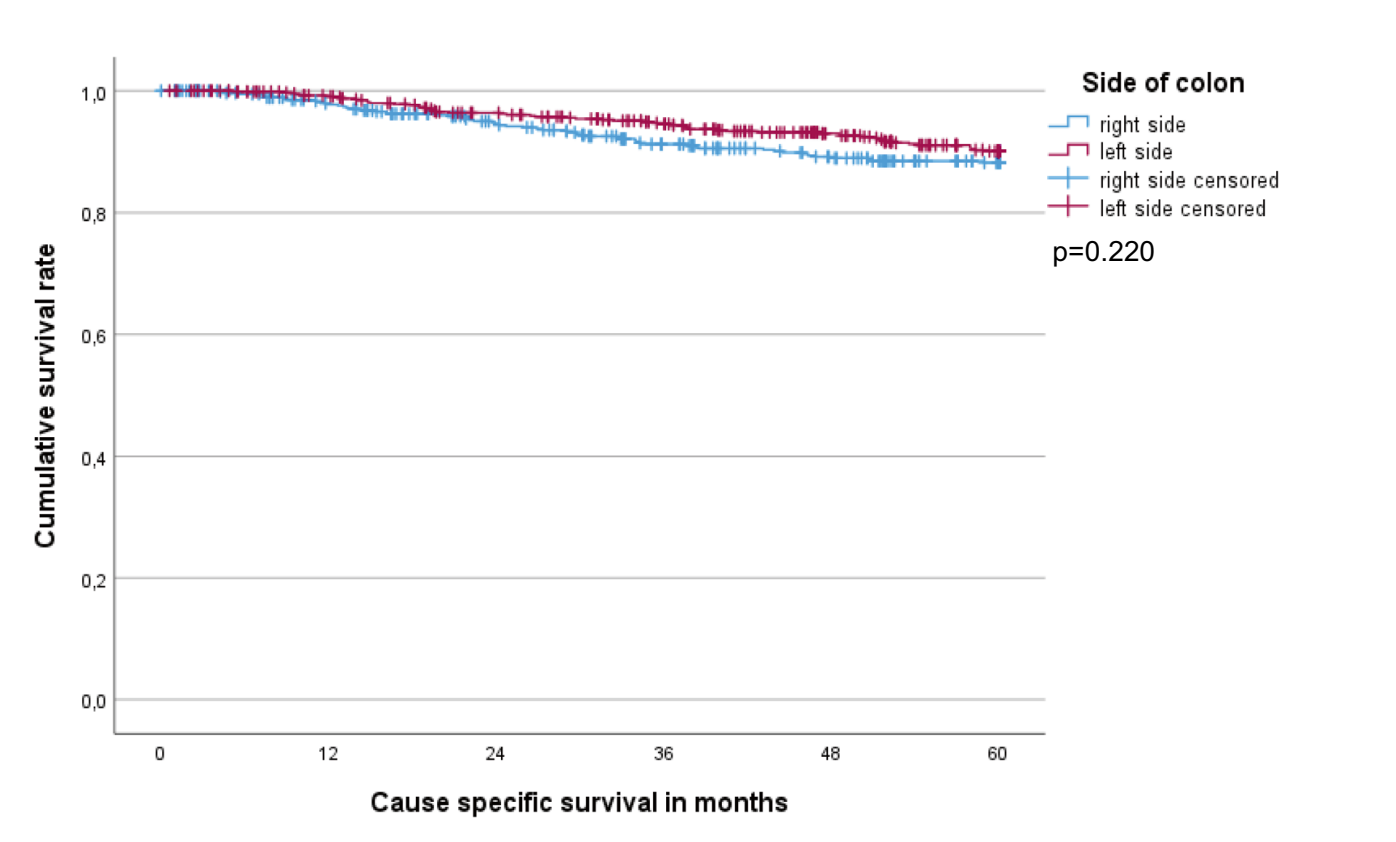
Five-year Cause-Specific Survival for stage I-III right- vs. left-sided colon cancer. Five-year cause-specific survival rates: right-sided colon (n = 555) 88.2%, left-sided colon (n = 667) 90.1% (p = 0.220). R0-resected patients only; in-hospital mortality excluded

**Fig. 3:**
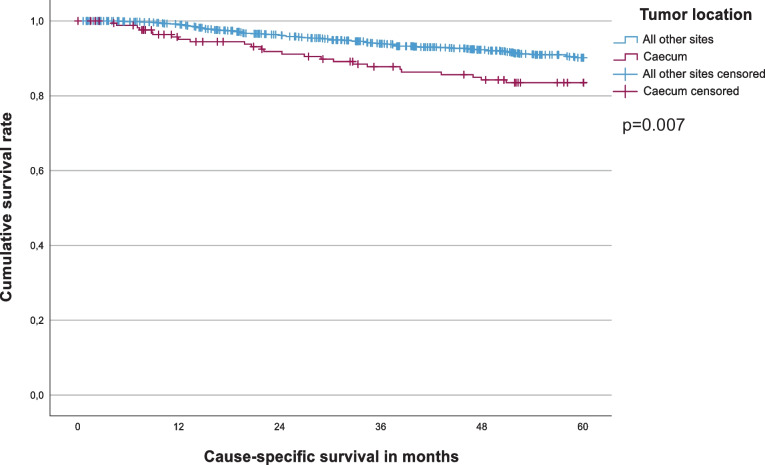
Five-year Cause-Specific Survival for stage I-III caecum cancer vs. all other colon cancer locations. Five-year cause-specific survival rate: caecum (n = 172) 83.5%, all other sites (n = 1,050) 90.2% (p = 0.007). R0-resected patients only; in-hospital mortality excluded

This group comprised 655 male and 567 female patients of a median age (range) of 70 (25–97) years. Follow-up was complete in 98.3% (21 patients lost) with a median time to the last follow-up (surviving patients) of 10.2 years. The stage distribution was as follows: stage I n = 316 (25.9%), stage II n = 537 (43.9%), stage III n = 369 (30.2%). We found 555 (45.4%) right- and 667 (54.6%) left-sided tumors with 172 (14.1%) caecal carcinomas in the first group. Tumor stages differed somewhat in various locations; however, stage III tumors were almost equally distributed (range 25.0%-33.3%; Table 1). We observed 115 events within the first 5 years of follow-up. The five-year CSS did not differ between the two sides (right-sided 88.2%, left-sided 90.1%, p = 0.220, Fig. 2), but tumors located in the caecum displayed a significantly worse prognosis (83.5% vs. 90.2% (all other tumor sites), p = 0.007, Fig. 3). Age, pT-category, pN-category, grading, preoperative CEA-level, lymphovascular invasion, vascular invasion, emergency operation and adjuvant chemotherapy were all significantly associated with 5-year CSS (Table 2). In the multivariate analysis, pT-category, pN-category, grading, vascular invasion, emergency operation, adjuvant chemotherapy, and caecal carcinoma remained independent factors (Table 3).

**Table 1:** Distribution of UICC-stages across the colonic locations

	**Stage I (%)**	**Stage II (%)**	**Stage III (%)**
Caecum	30 (17.4)	85 (49.4)	57 (33.1)
Ascendens	57 (26.4)	96 (44.4)	63 (29.2)
Hepatic flexure	12 (17.6)	39 (57.4)	17 (25.0)
Transversum	23 (23.2)	46 (46.5)	30 (30.3)
Splenic flexure	13 (18.1)	35 (48.6)	24 (33.3)
Descendens	14 (23.0)	29 (47.5)	18 (29.5)
Sigmoid	167 (31.3)	207 (38.8)	160 (30.0)
Total	316 (25.9)	537 (43.9)	369 (30.2)

**Table 2:** Five-year cause specific survival rates for stage I-III colon carcinoma

	**n (%)**	**Events**	**5y cause-specific survival in % [95% CI (%)]**	**p**
Total	1222	115	89.3 [87.3 … 91.3]	
Age^a^				0.027
<70	587 (48.0)	48	91.2 [88.8 … 93.6]	
≥70	635 (52.0)	67	87.2 [84.3 … 90.1]	
Sex				0.476
Male	655 (53.6)	58	89.6 [87.1 … 92.1]	
Female	567 (46.4)	57	88.8 [86.1 … 91.5]	
Side				0.22
Right	555 (45.4)	57	88.2 [85.3 … 91.1]	
Left	667 (54.6)	58	90.1 [87.7 … 92.5]	
Location^b^				
Caecum	172 (14.1)	25	83.5 [77.6 … 89.4] vs. 90.2 [88.2 … 92.2]	0.007
Ascendens	216 (17.7)	14	92.0 [87.9 … 96.1] vs. 88.7 [86.5 … 90.9]	0.147
Hepatic flexure	68 ( 5.6)	8	87.3 [79.1 … 95.5] vs. 89.4 [87.4 … 91.4]	0.43
Transversum	99 ( 8.1)	10	88.8 [82.3 … 95.3] vs. 89.3 [87.3 … 91.3]	0.812
Splenic flexure	72 ( 5.9)	4	93.3 [86.8 … 99.8] vs. 89.0 [87.0 … 91.0]	0.254
Descendens	61 ( 5.0)	4	93.0 [86.3 … 99.7] vs. 89.1 [87.1 … 91.1]	0.493
Sigmoid	534 (43.7)	50	89.4 [86.7 … 92.1] vs. 89.2 [86.7 … 91.7]	0.702
Time period				0.865
1995-2006	684 (56.0)	68	89.0 [86.5 … 91.5]	
2007-2018	538 (44.0)	47	89.8 [87.1 … 92.5]	
UICC-stage				<0.001
I	316 (25.9)	5	98.2 [96.6 … 99.8]	
II	537 (43.9)	31	93.2 [90.8 … 95.6]	
III	369 (30.2)	79	75.3 [70.6 … 80.0]	
pT-Category				<0.001
1	129 (10.6)	3	97.4 [94.5 … 100]	
2	236 (19.3)	4	98.0 [96.0 … 100]	
3	754 (61.7)	72	88.9 [86.5 … 91.3]	
4	103 ( 8.4)	36	58.3 [47.7 … 68.9]	
pN-Category				<0.001
0	853 (69.8)	36	95.1 [93.5 … 96.7]	
1	257 (21.0)	42	80.9 [75.6 … 86.2]	
2	112 ( 9.2)	37	62.4 [52.6 … 72.2]	
Number of harvested lymph nodes				0.301
< 12	285 (23.3)	22	90.9 [87.2 … 94.6]	
≥ 12	937 (76.7)	93	88.8 [86.6 … 91.0]	
Grading				<0.001
1+2	915 (74.9)	61	92.2 [90.2 … 94.2]	
3+4	307 (25.1)	54	80.3 [75.6 … 85.0]	
Mucinous carcinoma				0.129
No	1115 (91.2)	101	89.6 [87.6 … 91.6]	
Yes	107 ( 8.8)	14	85.1 [77.8 … 92.4]	
Preoperative CEA				0.002
Normal	946 (77.4)	76	90.8 [88.8 … 92.8]	
Elevated	276 (22.6)	39	83.8 [79.1 … 88.5]	
Lymphovascular infiltration				<0.001
No	971 (79.5)	60	92.9 [91.1 … 94.7]	
Yes	251 (20.5)	55	74.6 [68.7 … 80.5]	
Vascular infiltration				<0.001
No	1088 (89.0)	86	91.0 [89.2 … 92.8]	
Yes	134 (11.0)	29	74.6 [66.4 … 82.8]	
Emergency operation				<0.001
No	1083 (88.6)	90	90.6 [88.6 … 92.6]	
Yes	139 (11.4)	25	77.9 [70.1 … 85.7]	
Adjuvant chemotherapy				0.025
No	998 (81.7)	83	90.4 [88.4 … 92.4]	
Yes	224 (18.3)	32	84.7 [79.8 … 89.6]	
Length of specimen				0.294
< 20 cm	148 (13.8)	10	91.6 [86.5 … 96.7]	
≥ 20 cm	1074 (86.2)	115	88.9 [86.9 … 90.9]	

Missing values supplemented by multiple imputation: preoperative CEA, n = 6; lymphovascular invasion, n = 21; vascular invasion, n = 26; length of specimen, n = 24

*CI* confidence interval

^a^median 70 (range 25–97) years

^b^5-year CCS is compared vs. all other locations

**Table 3:** Cox regression analysis for 5-year cause-specific survival in stage I-III colon carcinoma

	**Hazard Ratio**	**95 % CI**	**p**
Age			
<70	Ref.		
≥70	1.139	0.748 … 1.736	0.544
Location			
All other sites	Ref.		
Caecum	1.645	1.039 … 2.606	0.034
pT-Category			<0.001
T1+2	Ref.		
T3	3.253	1.466 … 7.217	0.004
T4	10.852	4.538 … 25.950	<0.001
pN-Category			<0.001
N0	Ref.		
N1	4.238	2.516 … 7.137	<0.001
N2	8.364	4.623 … 15.130	<0.001
Grading			
1+2	Ref.		
3+4	1.516	1.017 … 2.259	0.041
Preoperative CEA			
Normal	Ref.		
Elevated	0.929	0.618 … 1.398	0.724
Lymphovascular infiltration			
No	Ref.		
Yes	1.255	0.795 … 1.981	0.724
Vascular infiltration			
No	Ref.		
Yes	1.95	1.235 … 3.079	0.004
Emergency operation			
No	Ref.		
Yes	1.651	1.031 … 2.644	0.037
Adjuvant chemotherapy			
No	Ref.		
Yes	0.385	0.234 … 0.634	<0.001

*CI* confidence interval

Analysis of prognostic factors was repeated for patients with stage III disease. Again, sidedness was not associated with prognosis, but carcinoma of the caecum remained as independent prognostic factor in the multivariate analysis as well as pT-category, pN-category, vascular invasion, and chemotherapy (Supplementary Tables 1 and 2). Regarding RFS, age and emergency operation emerged as independent prognostic factors, whereas neither sidedness nor any sublocation of the colon was associated with prognosis (Supplementary Tables 3 and 4).

### Stage IV tumors

**Fig. 4:**
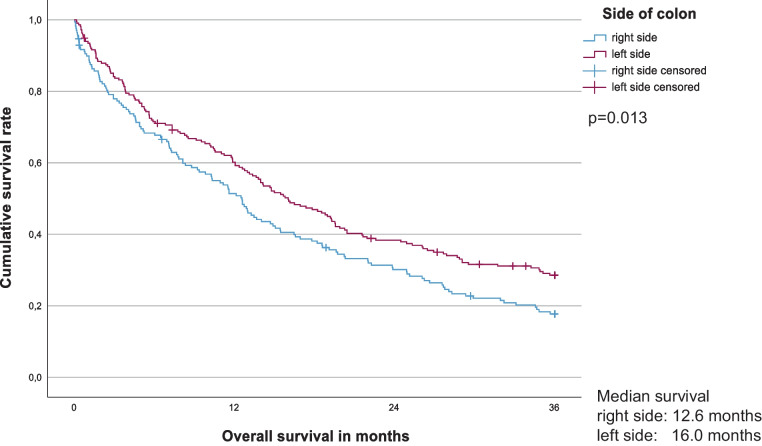
Three-year Overall Survival for stage IV right- vs. left-sided colon cancer. Three-year overall survival rate: right-sided colon (n = 169) 17.7%, left-sided colon (n = 215) 28.6% (p = 0.013)

**Fig. 5:**
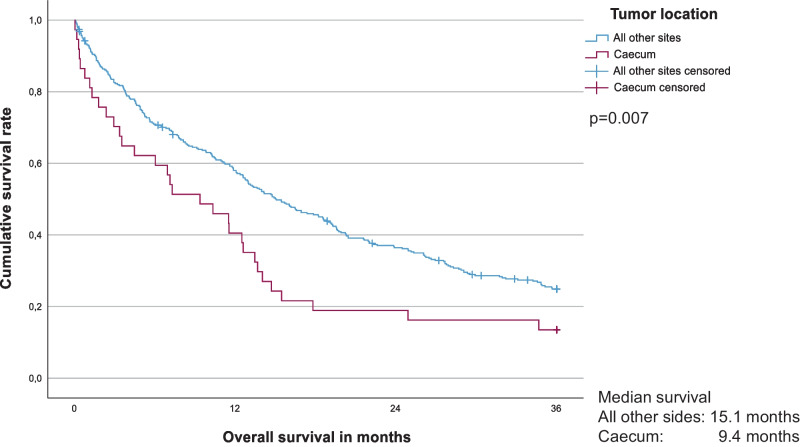
Three-year Overall Survival for stage IV caecum cancer vs. all other colon cancer locations. Three-year overall survival rate: caecum (n = 37) 13.5%, all other sites (n = 347) 24.9% (p = 0.007)

We identified 205 males and 179 females (median age: 71 (range 30–95) years) who presented with distant metastases at the time of tumor diagnosis. Eight patients (2.1%) were lost to follow-up. The median follow-up time for surviving patients was 6.3 years. Approximately half of the patients with stage IV disease had liver metastases only (n = 176, 45.8%), whereas in 111 (28.9%) patients multiple sites were involved. In 87 (22.6%) of these patients, complete removal of the primary tumor and the metastases was achieved (78 R0 resections (20.3%) and nine R1 resections (2.3%)). A further 206 (53.6%) patients underwent surgery for their primary tumor. In total, 287 events were observed during the first 3 years post-diagnosis. Right-sided tumors (n = 169 (44.0%)) had a significantly worse prognosis with 17.7% 3-year OS than left-sided tumors (n = 215 (56.0%)) with 28.6% 3-year OS (p = 0.013; Fig. 4). Patients with caecal carcinoma (n = 37 (9.6%)) had the worst prognosis (13.5% vs. 24.9% (all other locations) 3-year OS, p = 0.007; Fig. 5). The results differed also by time period with 3-year OS of 19.6% in 1995–2006 and 28.7% in 2007–2018 (p = 0.048). As expected, the best results were achieved after complete tumor removal (R0/R1-resections) with 3-year OS of 66.6% (vs. 10.7% for R2, p < 0.001). R0/R1-resections were achieved in n = 44/176 (25.0%) patients with liver metastases only compared to n = 33/97 (34.0%) patients with metastases at other single sites (mainly para-aortal lymph nodes, lung, or peritoneum) and n = 10/111 (9.0) patients with involvement of multiple sites. According to metastatic site, liver metastases showed a more favorable prognosis with a 30.5% 3-year OS (vs. 22.2% in other metastases and 14.5% in multiple sites (p < 0.001)). Systemic therapy was also associated with a better prognosis (32.7% 3-year OS vs. 17.7% (p < 0.001)) (Table 4). In multivariate analysis, T4-category, grading, removal of the primary tumor, R-classification, and systemic therapy were identified as independent prognostic factors (Table 5).

**Table 4:** Three-year overall survival rates for stage IV colon carcinoma

	**n (%)**	**Events**	**3y overall survival in % [95% CI (%)]**	**p**
Total	384	287	23.8 [19.5 … 28.1]	
Age^a^				<0.001
<71	181 (47.1)	125	29.5 [22.6 … 36.4]	
≥71	203 (52.9)	162	18.6 [13.1 … 24.1]	
Sex				0.082
Male	205 (53.4)	147	26.4 [20.1 … 32.7]	
Female	179 (46.6)	140	20.8 [14.7 … 26.9]	
Side				0.013
Right	169 (44.0)	136	17.7 [11.8 … 23.6]	
Left	215 (56.0)	151	28.6 [22.5 … 34.7]	
Location^b^				
Coecum	37 ( 9.6)	32	13.5 [ 2.5 … 24.5] 24.9 [13.9 … 35.9]	0.007
Ascendens	63 (16.4)	51	13.4 [ 4.4 … 22.4] 25.6 [20.7 … 30.5]	0.181
Hepatic flexure	38 ( 9.9)	32	15.8 [ 4.2 … 27.4] 24.7 [20.0 … 29.4]	0.146
Transversum	31 ( 8.1)	21	32.3 [15.8 … 48.8] 23.0 [18.5 … 27.5]	0.219
Splenic flexure	23 ( 6.0)	14	39.1 [22.6 … 55.6] 22.8 [18.4 … 27.1]	0.096
Descendens	24 ( 6.2)	15	37.0 [17.4 … 56.6] 22.9 [18.4 … 27.4]	0.318
Sigmoid	168 (43.8)	122	26.0 [19.3 … 32.7] 22.0 [16.3 … 27.7]	0.289
Time period				0.048
1995-2006	203 (52.9)	161	19.6 [14.1 … 25.1]	
2007-2018	181 (47.1)	126	28.7 [22.0 … 35.4]	
T-Category				<0.001
1+2^c^	14 ( 3.6)	8	40.2 [13.5 … 66.9]	
3	247 (64.3)	168	30.1 [24.2 … 36.0]	
4	123 (32.0)	111	9.4 [ 4.1 … 14.7]	
N-Category				0.005
0	115 (29.9)	90	20.5 [13.1 … 27.9]	
1	123 (32.0)	84	29.8 [21.6 … 38.0]	
2	146 (38.0)	113	21.1 [14.4 … 27.8]	
Grading				<0.001
1+2	223 (58.1)	155	29.0 [22.9 … 35.1]	
3+4	161 (41.9)	132	16.5 [10.6 … 22.4]	
Mucinous carcinoma				0.182
No	346 (90.1)	256	24.8 [20.1 … 29.5]	
Yes	38 (9.9)	31	14.1 [ 2.7 … 25.5]	
Pretherapeutic CEA				0.809
Normal	122 (31.8)	88	25.5 [17.7 … 33.3]	
Elevated	262 (68.2)	199	23.1 [18.0 … 28.2]	
Removal of primary tumor				<0.001
No	91 (23.7)	86	0	
Yes	293 (76.3)	201	30.6 [25.3 … 35.9]	
Emergency operation				< 0.001
No operation	21 (5.5)	17	0	
Emergency	87 (22.6)	76	12.6 [ 5.5 … 19.7]	
No emergency	276 (71.9)	194	28.6 [23.1 … 34.1]	
R-classification				<0.001
0+1	87 (22.6)	29	66.6 [56.6 … 76.6]	
2	297 (77.4)	258	10.7 [ 7.0 … 14.4]	
Metastatic site				<0.001
Liver	176 (45.8)	119	30.5 [23.6 … 37.4]	
Other	97 (25.3)	74	22.2 [13.8 … 30.6]	
Multiple	111 (28.9)	94	14.5 [ 7.8 … 21.2]	
Systemic therapy				<0.001
No	229 (59.6)	184	17.7 [12.6 … 22.8]	
Yes	155 (60.4)	103	32.7 [25.3 … 40.1]	

Missing values supplemented by multiple imputation: grading, n = 10; pretherapeutic CEA, n = 5

*CI* confidence interval

^a^median 71 (range 30 – 95) years

^b^3-year OS is compared vs. all other locations

^c^T1 and T2 were combined because of low numbers

**Table 5:** Cox regression analysis for 3-year overall survival in stage IV colon carcinoma

	**Hazard Ratio**	**95 % CI**	**p**
Age			
<71	Ref.		
≥71	0.988	0.753 … 1.296	0.988
Side			
Right	Ref.		
Left	1.086	0.831 … 1.421	0.545
Location			
All other sites	Ref.		
Caecum	0.836	0.551 … 1.268	0.399
Time period			
1995-2006	Ref.		
2007-2018	1.01	0.783 … 1.301	0.94
T-Category			0.014
T1+2	Ref.		
T3	1.291	0.621 … 2.684	0.493
T4	1.889	0.882 … 4.044	0.102
Grading			
1+2	Ref.		
3+4	1.573	1.219 … 2.031	<0.001
Removal of primary tumor			
No	Ref.		
Yes	0.557	0.417 … 0.743	<0.001
Emergency operation			0.139
No operation	Ref.		
Emergency	1.135	0.627 …2.054	0.675
No emergency	1.493	0.790 … 2.824	0.217
R-classification			
0/1	Ref.		
2	5.976	3.910 … 9.133	<0.001
Metastatic site			0.172
Liver	Ref.		
Other	1.247	0.890 … 1.746	0.199
Multiple	1.303	0.978 … 1.737	0.071
Systemic therapy			
No	Ref.		
Yes	0.43	0.320 … 0.578	<0.001

*CI* confidence interval

## Discussion

### Inhomogeneity of prognosis

Our study reveals that different parts of the colon are associated with different prognoses of cancer and that dividing the colon into right and left may be arbitrary. In the univariate analysis, no distinct pattern of prognosis was observed according to the colonic site. Especially in stages I-III, cancers of the ascending colon displayed an equally good prognosis as cancers of the splenic flexure and descending colon. Cancers of the right flexure and transverse colon resembled sigmoid cancers with respect to prognosis, and only caecal cancers stood out with a somewhat worse prognosis. The latter remained its prognostic value after adjusting for all potentially important factors in the multivariate Cox regression model. These findings are supported by the study of Benedix et al. who showed significant differences between the colonic subsites according to tumor stage, grading and the proportion of mucinous tumors [16]. The tumor stage did not follow a clear right/left pattern but showed, for instance, more advanced tumors in the caecum and less advanced tumors in the ascending colon. Similarly, Jess et al. observed a different pattern of prognosis for different tumor subsites in a nationwide cohort study that included 23,487 patients [17]. Tumors of the splenic flexure exhibited the highest relative mortality. Although this pattern does not fully comply with our results, it shows that differences exist that cannot be explained by the division of the colon into right and left.

### Sidedness in relation to stages I-III

The importance of sidedness in stage I-III CRC has not uniformly been reported in the literature [18–24]. Li et al. investigated a large patient series from the Surveillance, Epidemiology, and End Results (SEER) database [25]. In 238,826 patients, they found a better 5-year CSS in patients with right-sided tumors in stages I and II (HR 1.091 for left colon cancer). The clinical significance of this difference was low (88.9% vs. 87.0%, respectively). More importantly, they found a significant and clinically meaningful difference in survival in stage III with an HR of 0.799 for the left colon. Another study used the same data pool and reached similar results only 2 years later [26]. Interestingly, more T4- and N2- tumors were found in right-sided cancers, thus disadvantaging stages II and III. In contrast, other studies have explored the SEER database using propensity score-matched analysis [27, 28]. The better prognosis for left colon tumors, especially in stage III, disappeared after matching, suggesting external co-factors, such as fecal transit time, differences in the microbiome, and different clinical presentation. Moreover, the results for stage III may reflect the tumor response to systemic therapy because on the one hand adjuvant chemotherapy is recommended for stage III colon cancer, while on the other hand stage III cancer is prone to distant recurrence for which systemic therapy is the mainstay of treatment. Accordingly, Kennecke et al. demonstrated that sidedness was not a prognosticator for relapse-free survival in stage III tumors, however, once relapse occurred, the prognosis was inferior for right-sided cancers [29]. We did not find a significant difference between right- and left-sided stage III cancers, but adjuvant chemotherapy remained an independent prognostic factor in Cox regression analysis. Another large study explored the relevance of sidedness in a national database with regard to OS [30]. It used both direct and propensity score-matched analyses and found inferior outcomes for right-sided cancers in all tests. These differences disappeared only in patients with ≥ 22 harvested lymph nodes. The authors concluded that the adequacy of tumor resection might be decisive for the outcome differences in right- and left-sided cancers.

The largest study on this topic comprising some 1.4 million patients, was published by Petrelli et al. [31]. They performed a meta-analysis of 66 studies and provided HR for survival, showing inferior results for right-sided colon cancer across all stages. However, their ability to control for confounders was limited, and they only displayed results for OS. Because patients with right-sided cancer are, on average, older than those with left-sided cancer [16, 17, 25, 27], OS does not provide an unbiased measure of cancer survival, at least in stages I-III [20].

### Sidedness in relation to stage IV

Our multivariate analysis did not show a statistically significant difference in sidedness or time period in patients with stage IV disease. Instead, “classic” prognostic factors such as R-classification, T-category, grading, primary tumor resection, and application of systemic therapy remained in the model. This can be attributed mainly to inherent inhomogeneity of this patient group with very different tumor loads. Approximately one-quarter of patients underwent surgery with curative intention (R0/R1), indicating an oligometastatic state with a more favorable prognosis. Systemic therapy was also associated with better prognosis; thus, modern treatment options are clearly important prognostic factors in a non-selected stage IV patient cohort. We recently demonstrated that 30.6% of patients with synchronous metastases of CRC can undergo treatment with curative intent, with an OS of 53.0% at 5 years [32]. Similarly, Merkel et al. investigated the improvement in prognosis over time in a cohort of 937 patients who underwent primary tumor resection from the Erlangen Registry for Colorectal Carcinomas [33]. They found an increase in 2-year OS from 25.9% to 55.6% for the time periods 1985–1994 compared to 2005–2014. There were many more patients with unfavorable metastatic patterns in the right-sided tumor group, with 32.0% M1c tumors indicating peritoneal metastases (vs. 14.4% in left-sided tumors). In their multivariate Cox regression model, sidedness remained a prognostic factor for right-sided cancers with an HR of 1.2 (p = 0.012). The greater power and more homogenous patient group (excluding all patients with stage IV who could not undergo primary tumor resection as a positive selection) may explain this result.

The importance of sidedness as a prognosticator was established in patient cohorts including synchronous and metachronous metastases. These patients were usually not amenable to metastatectomy and were included in studies investigating new treatment agents, particularly cetuximab and bevacizumab [34–36]. In this scenario, RAS and BRAF mutation status has been proven to be of prognostic and predictive importance [37]. Several studies have shown that anti-EGFR therapy is less effective in metastases deriving from right-sided than from left-sided cancers even in RAS-wild-type tumors [2, 38]. Obviously, the more the patient selection process advances, the greater the importance of sidedness as a prognostic factor for metastatic CRC. However, the temporal and spatial multistep development of primary cancer and metastases regarding the evolution of cancer cell clones and their interaction with the tumor microenvironment are not yet fully understood [39, 40]. It may well be that the identification of all factors and their interaction in this complex tumorigenesis will result in individual tumor pathways that occur in different proportions at different sites in the colon. Some studies have suggested this direction [41–43].

The strength of our study is the large number of patients treated at a single institution with high-quality follow-up. The 5-year CSS rates compare favorably with those in the literature, with a rate of 89.3% for all stage I-III patients (88.2% for right-sided and 90.1% for left-sided tumors). For patients with stage III disease, the corresponding survival rates are 72.6% and 77.4%, respectively. Warschkow et al. calculated a 5-year CSS of 79.8% (right side) and 82.9% (left side) for all stage I-III patients from the SEER database and 63.6% (right side) and 64.6% (left side) for stage III patients [27]. A recent study by Merkel et al. showed a better survival rate in a stage I-III patient cohort in which prognostically favorable T1 carcinomas were excluded (89.3%) [44].

Our study harbors some limitations that need to be addressed. First, unlike large registry-based studies, we investigated a medium-sized patient cohort. Therefore, the results generated for the colon subsites must be interpreted with caution. Second, some prognostic factors of interest such as perineural invasion or RAS mutational status, were not completely documented or available over time and could therefore not be meaningfully investigated. This is counterbalanced by the overall high quality of the documented parameters and the low rate of missing data. Third, the type of adjuvant chemotherapy, generally based on 5-fluorouracil with a growing proportion of patients who received oxaliplatin, was not documented in our database. Hence the role of adjuvant chemotherapy could not be investigated in more detail. Fourth, in stage IV disease, we failed to identify sidedness as a prognostic factor even if we restricted our analysis to those patients who were not able to undergo metastatectomy (data not shown). Various factors including the long time period of observation, different tumor loads as mentioned above, and the evolving role of defering primary tumor resection [45] may have contributed to these results.

## Conclusions

In conclusion, our hypothesis was not supported. In patients without distant metastases, location in the caecum, but not sidedness per se, was identified as an independent prognostic factor. Similarly, the tumor side did not emerge as an independent prognostic factor in stage IV patients. Rather, it seems that different parts of the colon display tumor stages, molecular profiles, and other features in different proportions, and let sidedness appear as a surrogate for prognosis in some investigations. Therefore, further studies on the biological behavior of different colonic sites are warranted.

## Supplementary Information

Below is the link to the electronic supplementary material.

Supplementary file1 (DOCX 18 KB)

Supplementary file2 (DOCX 14 KB)

Supplementary file3 (DOCX 19 KB)

Supplementary file4 (DOCX 15 KB)

## Data Availability

Data will be provided by the authors upon reasonable request.
