# Supplementary material for: Sidedness is not a prognostic factor in an unselected cohort of patients with colon cancer but prognosis for caecal carcinoma is worse – A multivariate analysis of a large single institution database
Source: Int J Colorectal Dis. 2024 Feb 13;39(1):27. doi: 10.1007/s00384-023-04590-8 (PMC10864445; doi:10.1007/s00384-023-04590-8)
Supplement: Supplementary file 1 — Supplementary file1 (DOCX 18 KB) [file 384_2023_4590_MOESM1_ESM.docx]

Table 1

**Five-year cause specific survival rates for stage I-III colon carcinoma**

|  | **n (%)** | **Events** | **5y cause-specific survival in % [95% CI (%)]** | **p** |
| --- | --- | --- | --- | --- |
| Total | 369 | 79 | 75.3 [70.6 … 80.0] |  |
| Age^a^  <70  ≥70 | 180  189 | 38  41 | 77.0 [70.5 … 83.5]  73.9 [66.8 … 81.0] | 0.335 |
| Sex  Male  Female | 193  176 | 35  44 | 78.8 [72.5 … 85.1]  71.3 [64.0 … 78.6] | 0.063 |
| Side  Right  Left | 167  202 | 39  40 | 72.6 [65.2 … 80.0]  77.4 [71.1 … 83.7] | 0.253 |
| Location^b^  Caecum  Ascendens  Hepatic flexure  Transversum  Splenic flexure  Descendens  Sigmoid | 57  63  17  30  24  18  160 | 19  8  4  8  2  4  34 | 61.6 [47.9 … 75.3] 77.7 [72.6 … 82.8]  84.0 [73.8 … 94.2] 73.6 [68.3 … 78.9]  75.1 [53.7 … 96.5] 75.3 [70.4 … 80.2]  69.1 [51.1 … 87.1] 75.9 [71.0 … 80.8]  88.1 [72.0 … 100] 74.5 [69.6 … 79.4]  77.8 [58.6 … 97.0] 75.3 [70.4 … 80.2]  76.1 [69.0 … 83.2] 74.7 [68.2 … 81.2] | 0.005  0.088  0.751  0.478  0.147  0.882  0.632 |
| Time period  1995-2006  2007-2018 | 193  176 | 48  31 | 72.2 [65.5 … 78.9]  79.7 [73.2 … 86.2] | 0.253 |
| pT-Category  1  2  3  4 | 8  41  266  54 | 0  2  51  26 | 100  94.0 [86.0 … 100]  77.8 [72.3 … 83.3]  45.3 [30.8 … 59.8] | <0.001 |
| pN-Category  0  1  2 | -  257  112 | 42  37 | 80.9 [75.6 … 86.2]  62.4 [52.6 … 72.2] | <0.001 |
| Number of harvested lymph nodes  < 12  ≥ 12 | 66  303 | 11  68 | 79.3 [68.3 … 90.3]  74.5 [69.2 … 79.8] | 0.405 |
| Grading  1+2  3+4 | 239  130 | 37  42 | 81.5 [76.0 … 87.0]  64.2 [55.4 … 73.0] | <0.001 |
| Mucinous carcinoma  No  Yes | 334  35 | 69  10 | 76.2 [71.3 … 81.1]  65.9 [48.7 … 83.1] | 0.165 |
| Preoperative  CEA  Normal  Elevated | 248  121 | 49  30 | 76.9 [71.2 … 82.6]  72.3 [63.7 … 80.9] | 0.481 |
| Lymphovascular infiltration  No  Yes | 198  171 | 31  48 | 81.9 [76.0 … 87.8]  67.5 [59.9 … 75.1] | <0.001 |
| Vascular infiltration  No  Yes | 295  74 | 54  25 | 78.9 [73.8 … 84.0]  60.2 [47.7 … 72.7] | <0.001 |
| Emergency operation  No  Yes | 321  48 | 63  16 | 77.5 [72.6 … 82.4]  59.6 [43.5 … 75.7] | 0.002 |
| Adjuvant chemotherapy  No  Yes | 169  200 | 50  29 | 62.7 [54.3 … 71.1]  84.3 [79.0 … 89.6] | <0.001 |
| Length of specimen  < 20 cm  ≥ 20 cm | 45  324 | 6  73 | 83.2 [70.9 … 95.5]  74.1 [69.0 … 79.2] | 0.123 |

CI – confidence interval
