# Supplementary material for: Sidedness is not a prognostic factor in an unselected cohort of patients with colon cancer but prognosis for caecal carcinoma is worse – A multivariate analysis of a large single institution database
Source: Int J Colorectal Dis. 2024 Feb 13;39(1):27. doi: 10.1007/s00384-023-04590-8 (PMC10864445; doi:10.1007/s00384-023-04590-8)
Supplement: Supplementary file 2 — Supplementary file2 (DOCX 14 KB) [file 384_2023_4590_MOESM2_ESM.docx]

Table 2

**Cox regression analysis for 5-year cause-specific survival in stage III colon carcinoma**

|  | **Hazard Ratio** | **95 % CI** | **p** |
| --- | --- | --- | --- |
| Location  All other sites  Caecum | Ref.  2.136 | 1,230 … 3.711 | 0.007 |
| pT-Category  T1+2  T3  T4 | Ref.  4.880  11.847 | 1.169 … 20.364  2.722 … 51.573 | <0.001  0.030  <0.001 |
| pN-Category  N1  N2 | Ref.  2.031 | 1.261 … 3.272 | 0.004 |
| Grading  1+2  3+4 | Ref.  1.565 | 0.970 … 2.515 | 0.067 |
| Lymphovascular infiltration  No  Yes | Ref.  1.057 | 0.633 … 1.763 | 0.833 |
| Vascular infiltration  No  Yes | Ref.  2.206 | 1.310 … 3.717 | 0.003 |
| Emergency operation  No  Yes | Ref.  1.434 | 0.806 … 2.549 | 0.220 |
| Adjuvant chemotherapy  No  Yes | Ref.  0.308 | 0.192 … 0.494 | <0.001 |

CI – confidence interval
