# Supplementary material for: Sidedness is not a prognostic factor in an unselected cohort of patients with colon cancer but prognosis for caecal carcinoma is worse – A multivariate analysis of a large single institution database
Source: Int J Colorectal Dis. 2024 Feb 13;39(1):27. doi: 10.1007/s00384-023-04590-8 (PMC10864445; doi:10.1007/s00384-023-04590-8)
Supplement: Supplementary file 3 — Supplementary file3 (DOCX 19 KB) [file 384_2023_4590_MOESM3_ESM.docx]

Table 3

**Five-year disease-free survival rates for stage I-III colon carcinoma**

|  | **n (%)** | **Events** | **5y disease-free survival in % [95% CI (%)]** | **p** |
| --- | --- | --- | --- | --- |
| Total | 1222 | 365 | 69.4 [66.9 … 71.9] |  |
| Age^a^  <70  ≥70 | 587 (48.0)  635 (52.0) | 120  245 | 79.2 [75.9 … 82.5]  60.3 [56.4 … 64.2] | <0.001 |
| Sex  Male  Female | 655 (53.6)  567 (46.4) | 202  163 | 70.0 [66.5 … 73.5]  70.6 [66.9 … 74.3] | 0.469 |
| Side  Right  Left | 555 (45.4)  667 (54.6) | 166  199 | 69.4 [65.5 … 73.3]  69.5 [66.0 … 73.0] | 0.755 |
| Location^b^  Caecum  Ascendens  Hepatic flexure  Transversum  Splenic flexure  Descendens  Sigmoid | 172 (14.1)  216 (17.7)  68 ( 5.6)  99 ( 8.1)  72 ( 5.9)  61 ( 5.0)  534 (43.7) | 58  61  21  26  23  19  157 | 66.0 [58.9 … 73.1] 70.0 [67.3 … 72.7]  70.6 [64.3 … 76.9] 69.2 [66.3 … 72.1]  68.6 [56.4 … 78.8] 69.5 [66.8 … 72.2]  73.1 [64.3 … 81.9] 69.1 [66.4 … 71.8]  67.6 [56.6 … 78.6] 69.6 [66.9 … 72.3]  67.4 [55.2 … 80.0] 69.5 [66.8 … 72.2]  70.0 [66.1 … 73.9] 69.1 [65.6 … 72.6] | 0.148  0.606  0.744  0.439  0.753  0.764  0.554 |
| Time period  1995-2006  2007-2018 | 684 (56.0)  538 (44.0) | 203  162 | 70.2 [66.7 … 73.7]  68.4 [64.3 … 72.5] | 0.447 |
| UICC-stage  I  II  III | 316 (25.9)  537 (43.9)  369 (30.2) | 50  146  169 | 83.6 [79.5 … 87.7]  72.1 [68.2 … 76.0]  53.3 [48.2 … 58.4] | <0.001 |
| pT-Category  1  2  3  4 | 129 (10.6)  236 (19.3)  754 (61.7)  103 ( 8.4) | 17  49  237  62 | 86.3 [80.2 … 92.4]  78.5 [73.2 … 83.8]  67.9 [64.6 … 71.2]  39.0 [29.4 … 48.6] | <0.001 |
| pN-Category  0  1  2 | 853 (69.8)  257 (21.0)  112 ( 9.2) | 196  105  64 | 76.4 [73.5 … 79.3]  58.0 [51.9 … 64.1]  42.1 [32.9 … 51.3] | <0.001 |
| Number of harvested lymph nodes  < 12  ≥ 12 | 285 (23.3)  937 (76.7) | 89  276 | 68.6 [65.7 … 71.5]  69.7 [66.8 … 72.6] | 0.654 |
| Grading  1+2  3+4 | 915 (74.9)  307 (25.1) | 244  121 | 72.6 [69.7 … 75.5]  60.0 [54.5 … 65.5] | <0.001 |
| Mucinous carcinoma  No  Yes | 1115 (91.2)  107 ( 8.8) | 325  40 | 70.1 [67.4 … 72.8]  62.3 [53.1 … 71.5] | <0.001 |
| Preoperative CEA  Normal  Elevated | 946 (77.4)  276 (22.6) | 261  104 | 71.7 [68.8 … 74.6]  61.8 [55.9 … 67.7] | <0.001 |
| Lymphovascular infiltration  No  Yes | 971 (79.5)  251 (20.5) | 252  113 | 73.5 [70.8 … 76.2]  53.4 [44.6 … 62.2] | <0.001 |
| Vascular infiltration  No  Yes | 1088 (89.0)  134 (11.0) | 311  54 | 70.9 [68.2 … 73.6]  57.3 [48.5 … 66.1] | <0.001 |
| Emergency operation  No  Yes | 1083 (88.6)  139 (11.4) | 294  71 | 72.2 [69.5 … 74.9]  48.4 [40.0 … 56.8] | <0.001 |
| Adjuvant chemotherapy  No  Yes | 998 (81.7)  224 (18.3) | 299  66 | 69.4 [66.5 … 72.3]  69.8 [63.7 … 75.9] | 0.895 |
| Length of specimen  < 20 cm  ≥ 20 cm | 148 (13.8)  1074 (86.2) | 48  365 | 66.4 [58.6 … 74.2]  69.8 [67.1 … 72.5] | 0.548 |

CI – confidence interval

a - median 70 (range 25-97) years, b – 5-year CCS is compared vs. all other locations

Missing values supplemented by multiple imputation: preoperative CEA, n=6; lymphovascular invasion, n=21; vascular invasion, n=26; length of specimen, n= 24
