# Supplementary material for: Sidedness is not a prognostic factor in an unselected cohort of patients with colon cancer but prognosis for caecal carcinoma is worse – A multivariate analysis of a large single institution database
Source: Int J Colorectal Dis. 2024 Feb 13;39(1):27. doi: 10.1007/s00384-023-04590-8 (PMC10864445; doi:10.1007/s00384-023-04590-8)
Supplement: Supplementary file 4 — Supplementary file4 (DOCX 15 KB) [file 384_2023_4590_MOESM4_ESM.docx]

Table 4

**Cox regression analysis for 5-year disease-free survival in stage I-III colon carcinoma**

|  | **Hazard Ratio** | **95 % CI** | **p** |
| --- | --- | --- | --- |
| Age  <70  ≥70 | Ref.  1.749 | 1.378 … 2.220 | <0.001 |
| pT-Category  T1+2  T3  T4 | Ref.  1.373  3.145 | 1.029 … 1.832  2.131 … 4.642 | <0.001  0.031  <0.001 |
| pN-Category  N0  N1  N2 | Ref.  2.276  3,623 | 1.720 … 3.012  2.517 … 5.214 | <0.001  <0.001  <0.001 |
| Grading  1+2  3+4 | Ref.  1.104 | 0.857 … 1.423 | 0.443 |
| Preoperative CEA  Normal  Elevated | Ref.  1.004 | 0.857 … 1.423 | 0.975 |
| Lymphovascular infiltration  No  Yes | Ref.  1.179 | 0.893 … 1.557 | 0.245 |
| Vascular infiltration  No  Yes | Ref.  1.248 | 0.919 … 1.694 | 0.156 |
| Emergency operation  No  Yes | Ref.  1.680 | 1.282 … 2.202 | <0.001 |
| Adjuvant chemotherapy  No  Yes | Ref.  0.486 | 0.352 … 0.672 | <0.001 |

CI – confidence interval

Adjuvant chemotherapy was forced into the model because of its clear advantage shown in the Cox regression analysis done for the event “death with tumor”. Likewise, Side and Location were sequentially forced into the model, but did not show an independent association with disease-free survival.
